# Prediction of *TERT* mutation status in gliomas using conventional MRI radiogenomic features

**DOI:** 10.3389/fneur.2024.1439598

**Published:** 2024-07-26

**Authors:** Chuyun Tang, Ling Chen, Yifan Xu, Lixuan Huang, Zisan Zeng

**Affiliations:** Department of Radiology, The First Affiliated Hospital of Guangxi Medical University, Nanning, China

**Keywords:** glioma, magnetic resonance imaging, *TERT* promoter mutation, radiomics, machine learning

## Abstract

**Objective:**

Telomerase reverse transcriptase (*TERT*) promoter mutation status in gliomas is a key determinant of treatment strategy and prognosis. This study aimed to analyze the radiogenomic features and construct radiogenomic models utilizing medical imaging techniques to predict the *TERT* promoter mutation status in gliomas.

**Methods:**

This was a retrospective study of 304 patients with gliomas. T1-weighted contrast-enhanced, apparent diffusion coefficient, and diffusion-weighted imaging MRI sequences were used for radiomic feature extraction. A total of 3,948 features were extracted from MRI images using the FAE software. These included 14 shape features, 18 histogram features, 24 gray level run length matrix, 14 gray level dependence matrix, 16 gray level run length matrix, 16 gray level size zone matrix (GLSZM), 5 neighboring gray tone difference matrix, and 744 wavelet transforms. The dataset was randomly divided into training and testing sets in a ratio of 7:3. Three feature selection methods and six classification algorithms were used to model the selected features. Predictive performance was evaluated using receiver operating characteristic curve analysis.

**Results:**

Among the evaluated classification algorithms, the combination model of recursive feature elimination (RFE) with linear regression (LR) using six features showed the best diagnostic performance (area under the curve: 0.733, 0.562, and 0.633 in the training, validation, and testing sets, respectively). The next best-performing models were naive Bayes, linear discriminant analysis, autoencoder, and support vector machine. Regarding the three feature selection algorithms, RFE showed the most consistent performance, followed by relief and ANOVA. T1-enhanced entropy and GLSZM derived from T1-enhanced images were identified as the most critical radiomics features for distinguishing *TERT* promoter mutation status.

**Conclusion:**

The LR and LRLasso models, mainly based on T1-enhanced entropy and GLSZM, showed good predictive ability for *TERT* promoter mutations in gliomas using radiomics models.

## 1 Introduction

Gliomas are the most common and aggressive brain tumors originating from the glial cells. Owing to their rapid growth and invasive growth characteristics, gliomas are one of the most challenging types of brain cancer (1). The advances in molecular biology have enabled an in-depth understanding of the pathogenetic mechanisms of glioblastoma, including mutations in the telomerase reverse transcriptase *(TERT)* promoter. The replication of chromosomes during cell division entails the consumption of telomeric regions in each DNA replication cycle, eventually leading to cell division arrest (2–4). For unlimited proliferation of cancer cells, they must express telomerase reverse transcriptase (*TERT*) to maintain telomere length, preventing DNA shortening during replication, and achieving immortality (5). Several studies have reported an association between telomerase activation or increased *TERT* gene expression and the survival outcomes of gliomas. In *IDH1* wild-type glioblastomas with *MGMT* promoter methylation, individuals with conditions conducive to *TERT* promoter mutations exhibit better survival following standard radiotherapy and chemotherapy. Thus, the prognosis of *MGMT* promoter methylation depends on the *TERT* promoter mutation status (6).

Among patients with lower-grade gliomas (LGGs), those harboring *TERT* mutations have been found to exhibit a more favorable prognosis than those with wild-type *TERT* (7, 8). However, a considerable proportion of the LGG population shares similar genetic mutation features and survival characteristics with glioblastoma patients, and they may potentially progress to a more malignant state (9). A study by Killela et al. showed that *TERT* promoter mutations are very common in glioblastoma (GBM) and are associated with tumor subtypes with lower self-renewal ability of tumor cells. In addition, they also found that patients with primary GBM without *TERT* mutation survive longer than other patients with primary GBM (10). It is worth noting that mutations in the *TERT* promoter are linked to the prognosis and treatment resistance in patients with gliomas, indicating their significant impact on disease outcomes and therapeutic response (11, 12). Early studies have shown that cells lacking expression of human telomerase reverse transcriptase exhibit significantly reduced telomerase activity, i.e., attenuated tumorigenicity. Based on this, a novel telomerase inhibitor can be used in the treatment of glioma by combining its anti-tumor effect with radiotherapy and chemotherapy (13). Additionally, researchers have developed a new class of potent telomerase inhibitors that may enhance the sensitivity of conventional cytotoxic cancer therapies by targeting *TERT* (14). Due to the highly complex biological behavior and highly malignant nature of brain gliomas, the prognosis of these patients remains unsatisfactory even after comprehensive treatment (15). Therefore, identification of biomarkers that enable preoperative prognostic assessment is imperative to help guide postoperative treatment plans. This can help improve the therapeutic effectiveness and enhance survival outcomes. Currently, the primary method for detecting *TERT* promoter mutations in gliomas involves obtaining tumor tissue through biopsy or surgical resection. While this pathological examination method is accurate, it often entails lengthy surgeries. Additionally, detecting *TERT* promoter status can be challenging if the surgical scope is not precise or extensive enough. Furthermore, false-positive or false-negative test results are a concern. In contrast, preoperative MRI examinations allow for the acquisition of multi-dimensional, multi-parameter images of tumor tissue, enabling the formulation of treatment plans for patients with maximum accuracy. Therefore, radiomics, a field that extracts quantitative features from medical images, has emerged as a promising approach for non-invasive biomarker detection. By analyzing MRI images, radiomics can identify patterns and features that are associated with *TERT* promoter mutation status, enabling accurate prediction and improving patient outcomes. Since its formal proposal by Lambin et al. (16), radiomics has been widely applied in oncology research to aid diagnosis, prognostic prediction, and treatment decision-making in cancer patients (17, 18). It has been shown to be useful in the clinical management of patients with neurogliomas. Currently, most traditional radiomics approaches depend on individual sequences or algorithms to construct predictive models. To the best of our knowledge, in a study of Navodini et al. on data engineering-based glioma survival analysis, this study mainly focuses on using deep learning methods to extract features from MRI images to predict *IDH* mutation status (19), but it still has its limitations. The study did not use different feature selection and algorithms for model combination and comparison of their performance. Gabriele et al. conducted in-depth research on deep learning automatic tissue segmentation in patients with congenital or acquired brain anatomical malformations, realized automated brain tissue image segmentation, and reduced the workload of manual annotation. This undoubtedly demonstrates the great potential of deep learning in medical image analysis. But may have overlooked the potential value of biomarker data. Sasmitha et al. constructed a new framework in a study on the segmentation and classification of brain tumor MRI images using machine learning, 3D U-Net for segmentation and DenseNet-BC for classification, enabling a more comprehensive analysis of tumor features. Despite this, the intrinsic complexity of neural networks has the potential to pose challenges to the interpretability of decision-making processes. Based on the above discussion and findings, our study not only predicts *TERT* promoter mutations, but also uses a broader combination of machine learning algorithms. In addition, our method focuses more on the interpretability of the model, and in order to provide additional value to the clinic, we pay special attention to the influence of age as an important factor on the prediction of *TERT* mutations, which was not explored in detail in Navodini et al. Therefore, the aim of this study was to predict *TERT* promoter mutations in neurogliomas by using a combination of multiple MRI parameters. Further, we aimed to compare the performance of this approach with certain single-sequence predictive models. Toward this end, we integrated and analyzed MRI image features from T1-weighted contrast-enhanced (T1CE), diffusion-weighted imaging (DWI), and apparent diffusion coefficient (ADC) sequences. Three feature selection methods and six classification algorithms were employed to investigate whether preoperative multi-parameter MRI can differentiate *TERT* promoter mutation status in gliomas.

## 2 Methods

### 2.1 Technical contribution

Methodologically, we integrated the features of T1CE, DWI, and ADC sequences. This multi-parametric approach provides a comprehensive view of the tumor, utilizing different imaging modalities to capture a wide range of tumor features. 3,948 radiological features were subsequently extracted from the MRI images using FeAture Explorer Pro (FAE, V 0.5.7) software. These features include shape, histogram, gray run length matrix, gray dependency matrix, gray size region matrix (GLSZM), and wavelet transform. Using such a diverse set of features allows the model to capture complex patterns and changes in tumor images. Three feature selection methods, recursive feature elimination (RFE), Relief and analysis of variance (ANOVA), were then used to identify the most relevant features for model construction. This step is crucial for reducing the dimensionality of the feature space and focusing on the most informative features, thereby improving the performance and interpretability of the model. On this basis, we use 6 classification algorithms to model the selected features, including linear regression (LR), logistic regression via the least absolute shrinkage and selection operator (LR-Lasso), support vector machine (SVM), autoencoder (AE), linear discriminant analysis (LDA), and naive Bayes (NB). By comparing and combining different algorithms, the advantages of each algorithm are utilized to achieve the best prediction performance. This study places great emphasis on the interpretability of the model and ensures that the relationship between the selected features and the outcome (*TERT* promoter mutation status) can be easily understood and interpreted. This is critical for clinical acceptance and application. Different from previous studies, this method specifically takes age as an important factor in predicting *TERT* mutations. The analysis highlights the effect of age on mutation status, which adds an important dimension to the predictive model and enhances its clinical relevance. Besides, this retrospective study was approved by the Institutional Review Board. Review of patient images does not require patient approval or informed consent. However, informed consent was obtained for *TERT* promoter gene assessment during surgery.

### 2.2 Patients

**Figure 1:**
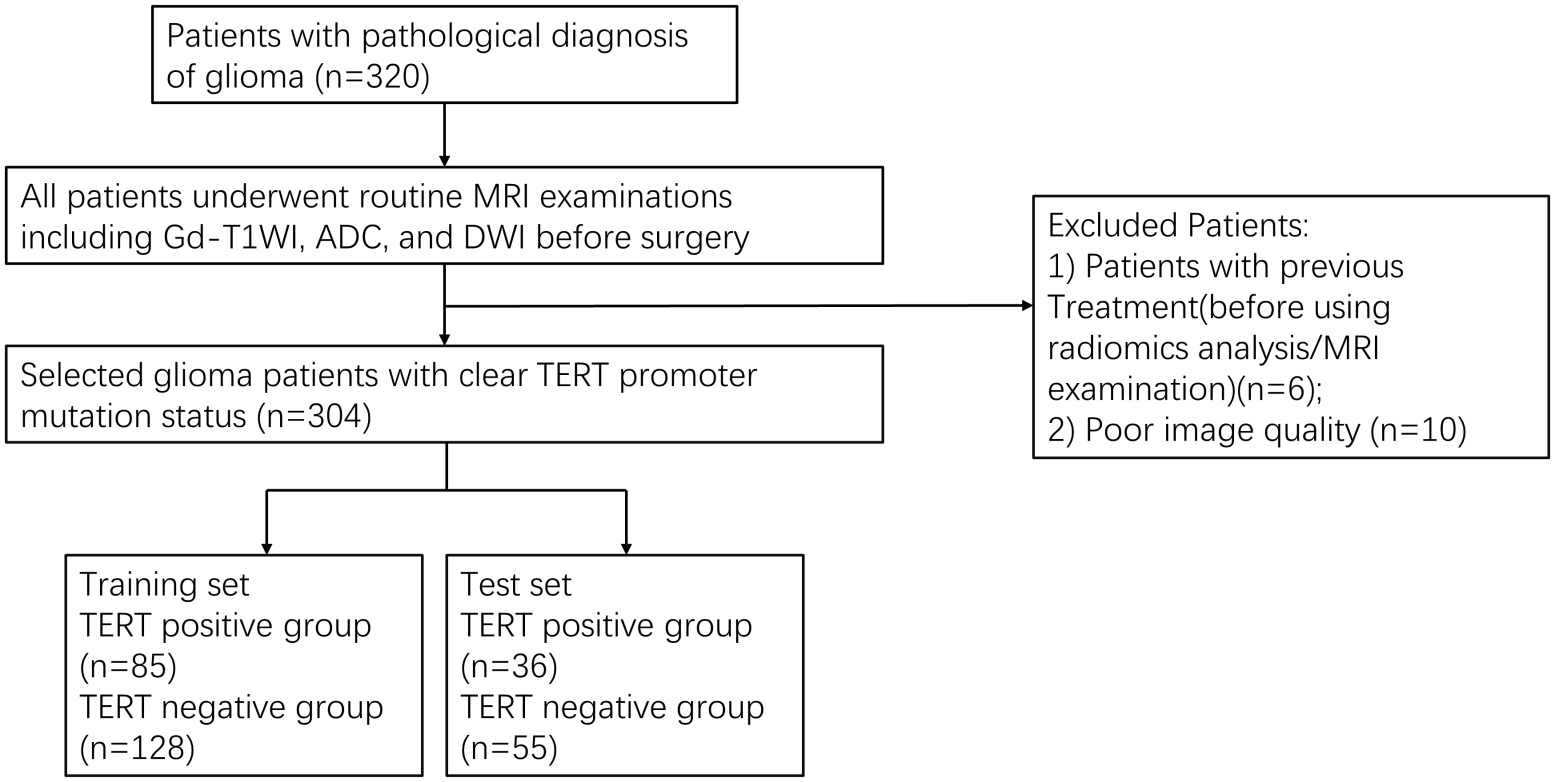
The patient selection flow chart.

We retrospectively searched our neuro-oncology database for patients with glioma between January 2019 and November 2023. The inclusion criteria were as follows: (1) patients diagnosed with grade 1–4 gliomas according to the 2021 WHO classification of central nervous system tumors; (2) availability of preoperative MRI images including T1CE, DWI, and ADC sequences with complete sequences and clear images; (3) complete molecular information of *TERT* obtained through the second-generation sequencing, including both C228T and C250T sites, along with clinical information; and (4) patients who had not received any treatment prior to their first baseline/diagnostic MRI that was used for radiomics analysis. A schematic illustration of the study design and patient-selection criteria is presented in Figure 1.

### 2.3 Assessment of the *TERT* promoter mutation status

All glioma specimens obtained through surgical resection or biopsy use the second-generation sequencing method for the identification of *TERT* promoter mutation status (20).

### 2.4 MRI protocol

All glioma patients underwent routine MRI examinations within 1 week before surgery. Four MR scanners (3.0T Canon; 1.5T Philips; 1.5T Siemens; 1.5T GE Premier) were used in this study population. The imaging sequences include axial T1C, DWI, and ADC; intravenous injection of gadolinium butanol (0.1 mmol/kg) was used for T1WI contrast-enhanced imaging. The other details of the MR scanning protocol are provided in Table 1.

**Table 1:** Magnetic resonance imaging acquisition details.

	3.0T Cannon	1.5T Philips	1.5T Siemens	1.5T GE
T1-CE				
TR/TE(ms)	447.4/5.5	2000/20	467/2.48	370/4.76
FOV	230 × 230	210 × 210	240 × 81.3	230 × 84.4
FA(°)	90	90	90	90
Matrix	224 × 224	212 × 150	256 × 256	256 × 153.6
Slice thickness/gap(mm)	5.5/1	6.0/1	5.0/1	5.0/1
DWI				
TR/TE(ms)	3362 × 90	2400/77	7500/94	400/73
FOV	240 × 240	230 × 230	230 × 100	230 × 100
FA(°)	90	90	90	90
Matrix	160 × 160	144 × 122	192 × 192	150 × 150
Slice thickness/gap(mm)	6.0/1	6.0/1	5.0/1	5.0/1

### 2.5 Image preprocessing and tumor segmentation

**Figure 2:**
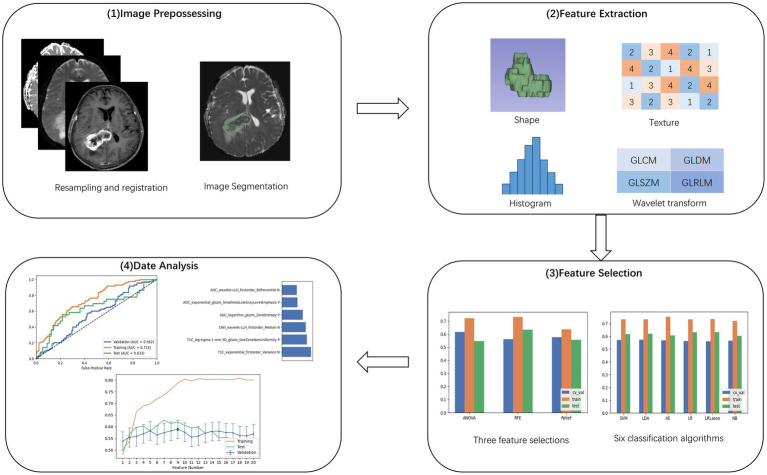
Procedure for image processing, extraction of radiomic features, and machine learning.

Preoperative MRI images of all patients, including T1CE, DWI, and ADC images, were resampled. This process was supervised by a neuroradiologist with 10 years of experience, who was blinded to the final diagnosis and molecular biomarker status. On the T1C images, the regions of interest (ROI) in the tumor were semi-automatically delineated and segmented layer by layer by the radiologist. Areas with hemorrhage, necrosis, and cystic changes were avoided during this procedure. After the completion of ROI segmentation, they were registered to the ADC and DWI images. An interclass correlation coefficient between 0.75 and 1.0 was deemed indicative of good consistency. All segmentation and registration tasks were performed using the 3D Slicer software (version 5.4.0).^1^ The steps of radiomics processing are illustrated in Figure 2.

### 2.6 Radiomics feature extraction

For each patient, a total of 3,948 radiomic features were extracted, comprising 1,316 features from each MRI sequence. These radiomics features were categorized into four major types: 14 shape features, 18 first-order metrics (such as the energy, entropy, mean, and kurtosis), and 75 textural features including 24 gray-level co-occurrence matrix, 14 gray-level dependence matrix, 16 gray-level run length matrix, 16 gray-level size zone matrix (GLSZM), and 5 neighborhood gray-tone difference matrix. Furthermore, the PyRadiomics database employs wavelet transformation to decompose the texture features extracted from segmented images into different scales and orientations. This approach generates various combinations of wavelet transformation series, including HHH, HHL, HLL, HLH, LLL, LLH, LHL, and LHH, to comprehensively capture the multi-scale and multi-directional characteristics of the images. From each decomposition, three types of texture features were extracted and 8 decompositions were obtained at each wavelet filtering stage. Finally, wavelet transformation results were obtained for 744 features. The specific feature attributes are shown in Table 2.

**Table 2:** Composition and details of radiomic feature attributes.

Feature classifier	Feature parameters (*n* = 1,316)
Shape feature (*n* = 14)	Elongation, flatness, least axis length, major axis length, maximum 2D diameter column, maximum 2D diameter row, maximum 2D diameter slice, maximum 3D diameter, mesh volume, minor axis length, sphericity, surface area, surface volume ratio, voxel volume
Histogram feature (*n* = 18)	P10, P90, interquartile range, energy, entropy, skewness, kurtosis, maximum, minimum, mean, mean absolute, deviation, median, total energy, uniformity, variance, range, robust mean absolute deviation, root mean squared
Texture feature (*n* = 75)	Gray-level co-occurrence matrix, GLCM (*n* = 24); gray-level dependence matrix, GLDM (*n* = 14); gray-level run length matrix, GLRLM (*n* = 16); gray-level size zone matrix, GLSZM (*n* = 16); neighborhood gray-tone difference matrix, NGTDM (*n* = 5)
Wavelet transform (*n* = 744)	Wavelet filtering produces eight decompositions per stage. In the three dimensions, all feasible combinations of high-pass or low-pass filters (HHH, HHL, HLL, HLH, LLL, LLH, LHL, and LHH)

### 2.7 Models establishment and statistical analysis

The datasets were randomly split into two groups: a training set and a test set, with a ratio of 7:3. Two hundred and thirteen cases were selected as the training dataset (85 *TERT* mutant and 128 *TERT* wild-type), and an additional 91 cases were selected as the independent test dataset (36 *TERT* mutant and 55 *TERT* wild-type). For each patient, 3,948 features were extracted. To address the imbalance in the training dataset, samples were augmented by random repetition to achieve a balanced distribution of positive and negative samples. The feature matrix was normalized, with each feature vector subtracted by its mean and divided by its standard deviation, resulting in a normalized vector with zero mean and unit standard deviation. Given the high dimensionality of the feature space, the Pearson correlation coefficient (PCC) was calculated between pairs of features. If the PCC value exceeded 0.990, one of the features was eliminated from the pair, reducing the feature space dimensionality to maintain feature independence. Before building the model, ANOVA was performed to select features and evaluate the significance of the relationship between features and labels by computing the *F*-value. Subsequently, a multilayer perceptron (MLP), also referred to as autoencoder (AE), was used as the classifier. The MLP is a neural network based on multiple hidden layers that learn the mapping from input features to labels. We employed one hidden layer with 100 hidden units, a linear unit activation function, Adam optimizer with a learning rate of 0.001. A 10-fold cross-validation was conducted on the training dataset to determine the maximum number of features required in the model. This was done to assess the performance of the model constructed on the validation set. The predictive capability of the model was assessed using receiver operating characteristic (ROC) curve analysis. The optimal Youden index threshold was used to calculate the accuracy, sensitivity, specificity, positive predictive value (PPV), and negative predictive value (NPV) of the model. Bootstrap estimation with 1,000 iterations was used to compute 95% confidence intervals for all metrics. All of these procedures were carried out using the FeAture Explorer Pro (FAE, V 0.5.7) Python package (version 3.7.6).

Subsequently, we employed combinations of any two feature selection methods among ANOVA, Relief, and RFE for feature screening and optimization. The *F*-value was calculated to assess the association between radiomics features and the label (*TERT* mutation status) in preparation for the subsequent model construction. A specific number of features were ranked based on their respective *F*-values. The features were selected based on the previously set *F*-values. Then, the selected features were modeled using LR, LR-Lasso, SVM, AE, LDA, and NB. Finally, we determined the hyperparameters used to build the model and chose 10-fold cross-validation. Based on the validation dataset in the constructed model, the hyperparameters according to the cross-validation results were used to optimize the model and assess its performance (Table 3).

**Table 3:** Characteristics of patients in the training set and test set.

Characteristics	Training set (*n* = 213)			Test set (*n* = 91)			
	*TERT*-mt	*TERT*-wt		*TERT*-mt	*TERT*-wt	*p*	*p*
Age (mean ± SD)	48.56 ± 14.22	34.78 ± 18.07	<0.001	48.75 ± 13.11	36.98 ± 19.44	0.002	0.548
<60	69	117	0.028	36	79	0.039	<0.001
≥60	16	11		10	6		
Sex			0.083			0.953	0.707
Male	59	84		26	30		
Female	26	44		10	25		
*TERT*	85	128	<0.001	36	55	<0.001	<0.955

TERT-mt telomerase reverse transcriptase mutant-type, TERT-wt telomerase reverse transcriptase wild-type.

In addition, the overall predictive performance of the model in the training, validation, and test sets was assessed using ROC curve analysis. Additionally, bootstrap estimation with 1,000 reiterations was employed to estimate the 95% confidence interval. Clinical features of the patients were analyzed using the Mann–Whitney U test for continuous variables and the chi-square test for categorical variables. All aforementioned descriptive statistical operations were conducted using SPSS 27.0 software. *p*-values <0.05 were considered indicative of statistical significance.

## 3 Results

### 3.1 Patient characteristics

Sixteen cases were excluded from the analysis, including 6 cases with a history of surgery, radiotherapy, or steroid therapy, and 10 cases due to poor MRI image quality. Finally, 304 glioma patients (143 males and 70 females) were included in this study. The average age of the patients was 40.94 ± 17.08 years. There were 121 cases (39.80%) with *TERT* promoter mutations and 183 cases (60.20%) without *TERT* promoter mutations. In the training dataset, there was a significant difference in age between the *TERT* subgroups (*p* < 0.001). A similar trend was observed in the validation dataset (*p* = 0.002). These findings indicated significant age differences among the *TERT* subgroups across different datasets and analytical settings. Furthermore, we divided the cases in the training and test sets into two groups: younger patients (age < 60 years) and older patients (age ≥ 60 years). Older patients were significantly more likely to have *TERT* promoter mutations in both sets (*p* = 0.028 and *p* = 0.039, respectively). Moreover, the *p*-value between the two sets was <0.001, indicating a significant difference in age distribution between these two sets. However, in both the training and test datasets, in the *TERT* subgroups, the difference in sex distribution was not statistically significant (*p* > 0.05 for all). This indicated a relatively balanced sex distribution across the different *TERT* subgroups in these two datasets.

### 3.2 Model validation and model comparison

We established and compared all radiomics models using FAE software, evaluated multiple selection thresholds to determine the optimal configuration for all radiomics features, and provided a detailed description of the ROC curve analysis results in both the training and test sets (Table 4).

**Table 4:** Performance analysis of each model in predicting *TERT* mutation status.

Feature set	AUC	95%CI	ACC	YI	Sen	Spe	PPV	NPV
Zscore_PCC_RFE_6_LR	0.633	[0.510–0.757]	0.692	0.337	0.556	0.782	0.625	0.729
Zscore_PCC_RFE_6_LRLasso	0.633	[0.508–0.757]	0.692	0.337	0.556	0.782	0.625	0.729
Zscore_PCC_RFE_5_LR	0.628	[0.509–0.748]	0.681	0.223	0.278	0.946	0.769	0.667
Zscore_PCC_RFE_7_NB	0.638	[0.506–0.760]	0.659	0.273	0.528	0.746	0.576	0.707
Zscore_PCC_RFE_4_LR	0.622	[0.503–0.741]	0.626	0.257	0.639	0.618	0.523	0.723
Zscore_PCC_RFE_6_LDA	0.620	[0.493–0.747]	0.659	0.312	0.639	0.673	0.561	0.740
Zscore_PCC_Relief_10_AE	0.619	[0.501–0.737]	0.560	0.225	0.861	0.364	0.470	0.800
Zscore_PCC_Relief_12_NB	0.617	[0.500–0.735]	0.571	0.224	0.806	0.418	0.475	0.767
Zscore_PCC_Relief_11_AE	0.6144	[0.492–0.735]	0.670	0.234	0.361	0.873	0.650	0.676
Zscore_PCC_Relief_2_AE	0.603	[0.484–0.721]	0.571	0.233	0.833	0.400	0.476	0.786
Zscore_PCC_Relief_11_SVM	0.590	[0.470–0.710]	0.528	0.170	0.861	0.309	0.449	0.773
Zscore_PCC_Relief_11_LR	0.59	[0.463–0.715]	0.6813	0.3	0.5	0.8	0.621	0.710
Zscore_PCC_ANOVA_3_NB	0.588	[0.462–0.714]	0.659	0.312	0.639	0.673	0.561	0.740
Zscore_PCC_ANOVA_11_AE	0.587	[0.461–0.713]	0.659	0.244	0.444	0.800	0.593	0.688
Zscore_PCC_ANOVA_4_NB	0.584	[0.458–0.710]	0.648	0.274	0.583	0.691	0.553	0.717
Zscore_PCC_ANOVA_11_NB	0.572	[0.445–0.698]	0.648	0.236	0.472	0.764	0.567	0.689
Zscore_PCC_ANOVA_12_NB	0.571	[0.445–0.698]	0.648	0.255	0.528	0.727	0.559	0.702
Zscore_PCC_ANOVA_10_NB	0.569	[0.442–0.696]	0.637	0.237	0.528	0.709	0.543	0.696

AUC, area under the curve; YI, Youden index; Acc, accuracy; Sen, sensitivity; Spe, specificity; PPV, positive predictive value; NPV, negative predictive value.

Furthermore, a comprehensive strategy was employed for feature selection. Specifically, we systematically paired the RFE, ANOVA, and Relief feature selectors randomly and thoroughly. These pairings were combined with six different classification algorithms, including LR, LRLasso, NB, LDA, AE, and SVM, to construct multiple composite models. Based on determining the maximum number of features, the range of feature selection was further refined using the “one standard error” criterion to obtain the optimal feature combinations. This integrated approach was employed to fully leverage the strengths of each feature selector and classification algorithm, thereby enhancing the performance and generalizability of the model. The following results were obtained after integrating features, building models, and conducting meticulous selection.

**Figure 3:**
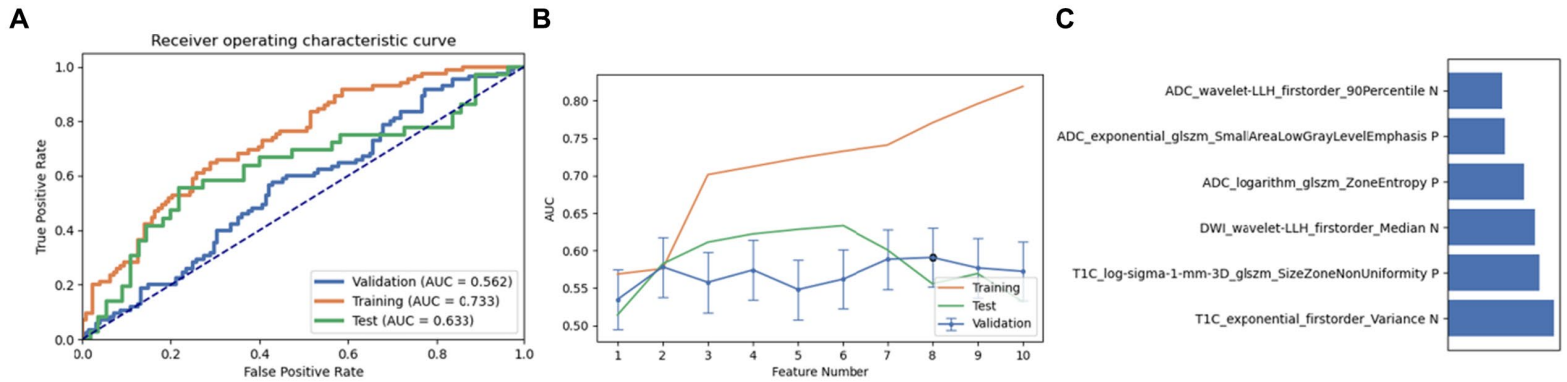
Performance of the model derived from recursive feature elimination (RFE) combined with Linear Regression (LR): **(A)** ROC curves for the validation set, training set, and test set; **(B)** Following the “one standard error” principle, the maximum number of features in the model was reduced to 6; **(C)** Ranking of omics feature contributions from the best model derived from the combination of RFE and LR.

Firstly, we chose a combination of RFE and ANOVA as the feature selection method. After screening and analysis, we identified a feature set containing 6 key features in LR. This feature combination showed a good performance in the cross-validation set, training set, and test set (AUC: 0.562, 0.733, and 0.633, respectively). Upon further ranking these features, the exponential first-order variance of T1C emerged as the most crucial and influential feature in this model (Figure 3).

**Figure 4:**
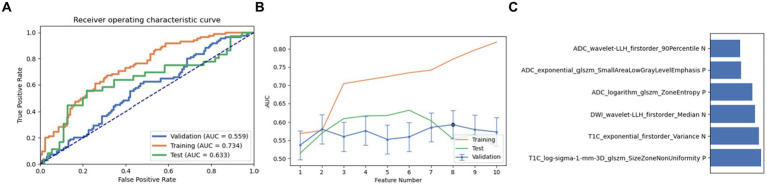
Performance of the model derived from recursive feature elimination (RFE) combined with logistic regression via the least absolute shrinkage and selection operator (LRLasso): **(A)** ROC curves for the validation set, training set, and test set; **(B)** Following the “one standard error” principle, the maximum number of features in the model was reduced to 6; **(C)** Ranking of omics feature contributions from the best model derived from the combination of RFE and LRLasso.

Next, a combination of Relief and RFE was selected for feature selection. After careful screening and analysis, we identified a feature set comprising 12 key features in LRLasso. This feature combination showed a good performance in the cross-validation set, training set, and test set (AUC: 0.559, 0.734, and 0.633, respectively). On further ranking these features, the size zone non-uniformity of log-sigma-1 mm-3D glszm of T1C emerged as the most crucial and influential feature in this model (Figure 4).

**Figure 5:**
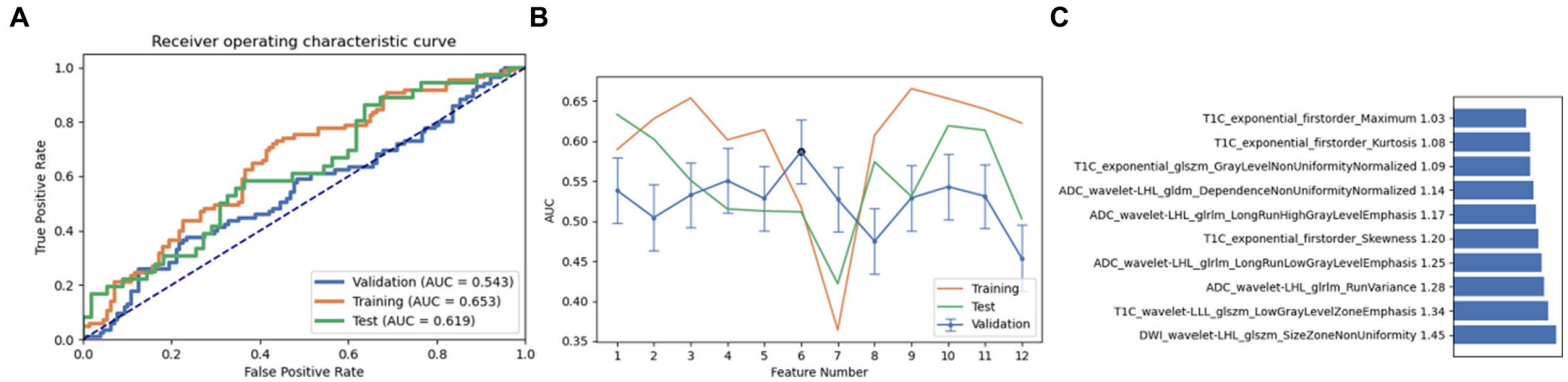
Performance of the model derived from relief combined with auto encoder (AE): **(A)** ROC curves for the validation set, training set, and test set; **(B)** Following the “one standard error” principle, the maximum number of features in the model was reduced to 12; **(C)** Ranking of omics feature contributions from the best model derived from the combination of relief and AE.

**Figure 6:**
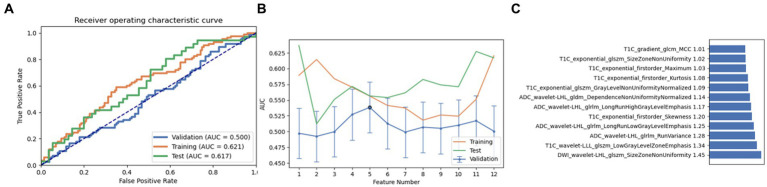
Performance of the model derived from relief combined with Naive Bayes (NB): **(A)** ROC curves for the validation set, training set, and test set; **(B)** Following the “one standard error” principle, the maximum number of features in the model was reduced to 12; **(C)** Ranking of omics feature contributions from the best model derived from the combination of relief and NB.

Finally, a combination of ANOVA and Relief was employed as the third feature selection method. Through screening and analysis, we identified a feature set containing 10 key features in AE. This feature combination achieved AUC values of 0.543, 0.653, and 0.619 in the cross-validation set, training set, and test set, respectively. Upon ranking, the wavelet-LHL glszm size zone non-uniformity of DWI was identified as the most influential feature (Figure 5). It is noteworthy that on setting the maximum feature count to 12, the naive Bayes model also achieved near-optimal AUC values (0.500, 0.621, and 0.617 in the cross-validation set, training set, and test set, respectively). The most contributing feature in this pipeline was again identified as the wavelet-LHL glszm size zone non-uniformity of DWI (Figure 6).

**Figure 7:**
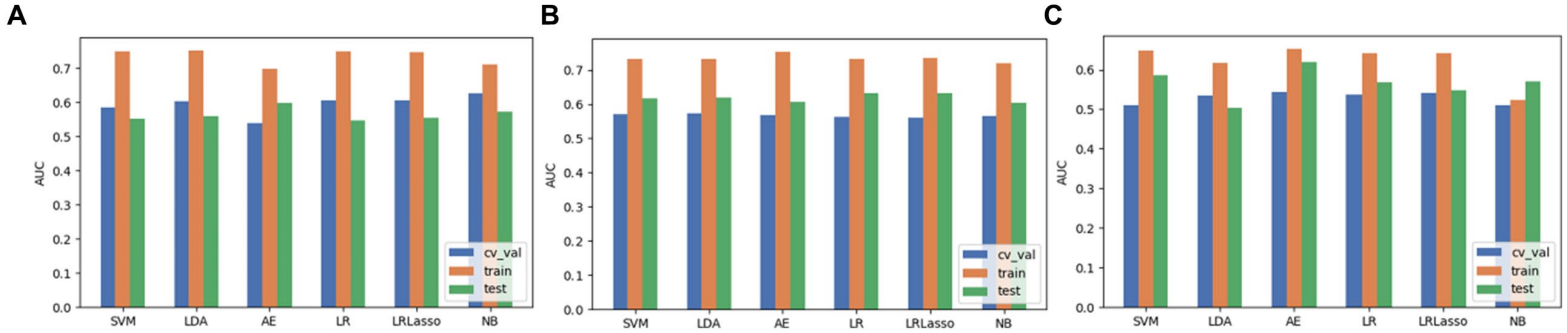
The performance evaluation of models when 3 feature selection methods (including ANOVA, RFE and relief) were integrated with 6 algorithms. **(A)** ANOVA. **(B)** RFE. **(C)** Relief.

**Figure 8:**
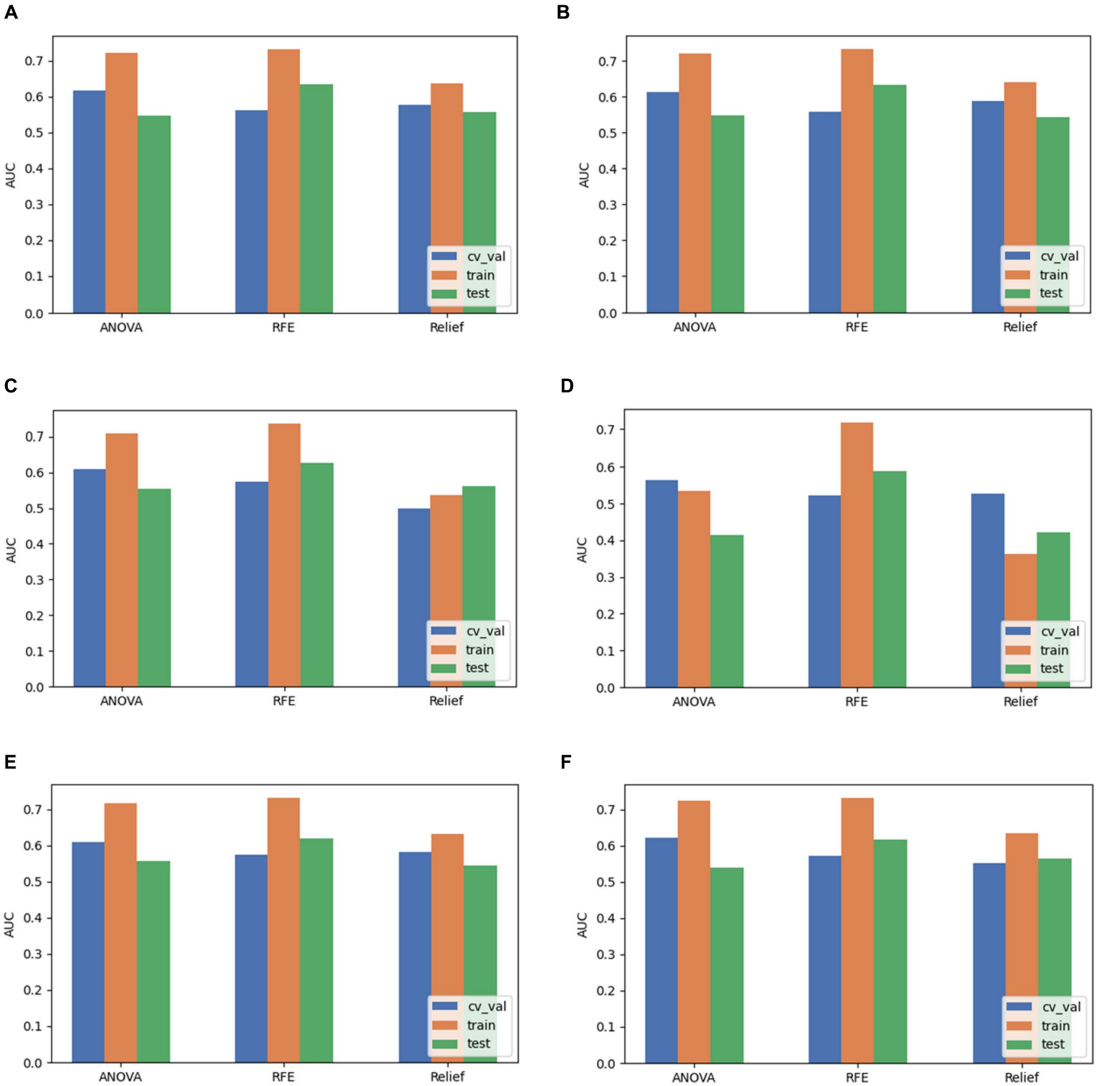
Comparison of performance when pairing LR, LRLasso, NB, AE, LDA, and SVM classification algorithms with three feature selection methods. **(A)** LR. **(B)** LRLasso. **(C)** NB. **(D)** AE. **(E)** LDA. **(F)** SVM.

Based on the aforementioned experimental results, we comprehensively evaluated the omics models constructed using three feature selection methods and six classification algorithms. DeLong nonparametric test was used for model comparison. Among the three feature selection methods, the omics model trained with RFE as a feature selector has significant consistency and stability. Specifically, the AUC values of the model on the validation set, the training set, and the test set were 0.56, 0.73, and 0.63. In contrast, the highest AUC values of ANOVA on the validation, training, and test sets were 0.59, 0.79, and 0.59. The highest AUC values of relief on the validation, training, and test sets were 0.54, 0.65, and 0.619. In the comparison of these three, we can clearly see that RFE has superior performance as a feature selector (Figure 7). In addition, the models constructed by RFE in conjunction with the above six classification algorithms did not show significant differences in predicting *TERT* mutation status in glioma patients. There was also no significant difference in the ability of RFE to differentiate *TERT* subtypes in glioma patients compared to ANOVA and Relief. Among the six classification algorithms, LR has the highest classification performance, followed by LRLasso (both AUC > 0.630). Furthermore, LR and LRLasso did not differ significantly in their ability to identify *TERT* subtypes in glioma patients; The highest AUC value of LDA can reach 0.62, which also shows good classification performance. The classification performance of AE is second to that of LDA, and the best AUC value of AE is 0.619; The AUC value of NB was almost the same, 0.617. As for SVM, its performance is less satisfactory, only 0.59 (Figure 8). A DeLong non-parametric test was used for model comparison. Among the six classification algorithms, LR demonstrated the highest performance, closely followed by LRLasso (AUC >0.630 for both). Moreover, there was no significant difference between the ability of LR and LRLasso to identify *TERT* subtypes in glioma patients. Among the three feature selection methods, the omics model trained using RFE as the feature selector exhibited notable consistency and stability. Specifically, this model achieved AUC values exceeding 0.6 on the validation set, training set, and test set, further demonstrating its superior performance. Additionally, the model constructed by combining RFE with the aforementioned six classification algorithms showed no significant difference in predicting *TERT* mutation status in glioma patients. When compared with ANOVA and Relief, RFE also did not exhibit significant differences in its ability to differentiate *TERT* subtypes in glioma patients.

## 4 Discussion

In this study, we investigated the potential of radiomics models, combined with routine MRI sequences (T1C, DWI, and ADC), in predicting the *TERT* mutation status of glioma patients. In this study, *TERT* promoter mutations were identified in 121 out of 304 glioma patients, accounting for 39.80% of cases. There was a significant difference in age distribution across *TERT* subgroups, but no significant difference in sex distribution. For predicting the *TERT* promoter mutation status in glioma patients, we utilized a combination of feature selection methods, including ANOVA, Relief, and RFE, along with classification algorithms such as LR, LRLasso, SVM, AE, LDA, and NB. Among these methods, RFE was the most effective feature selector for predicting *TERT* promoter mutations. LR and LRLasso were identified as the top-performing classifiers when assessed individually with these feature selectors, both achieving AUC values above 0.600.

Previous studies have found a positive correlation between *TERT* promoter mutation status and age at glioma diagnosis. The frequency of *TERT* promoter mutations increases with age (21). Similarly, Kim et al. identified older age as a risk factor for *TERT* promoter mutations in diffuse gliomas (22). Therefore, our results may enhance our understanding of glioma pathogenesis.

With the rapid advances in artificial intelligence technology, radiomics has shown potential as a bridge linking radiological images with tumors. It can convert images into high-dimensional data, thereby providing support for clinical treatment decisions (23). Previous studies have mainly focused on the use of single radiomics models. Tian et al. used an SVM model to classify low-grade gliomas from high-grade gliomas, achieving an AUC value of 0.987 (18). Fukuma et al. constructed a linear SVM model based on convolutional neural networks using MRI images of 164 patients with grade II/III gliomas to predict *TERT* promoter mutations (AUC: 0.82) (24). Although the aforementioned studies have yielded satisfactory results, they only analyzed the diagnostic performance of individual radiomics models without comparing them with other radiomics models. This is a major limitation in furthering the understanding and application of radiomics models.

Another study conducted radiomic feature analysis using T1CE and T2-weighted images of 83 patients with low-grade gliomas. They employed Lasso feature selection and built models including SVM, RF, and Adaboost. All three models demonstrated good predictive performance for the *TERT* promoter mutation status in low-grade gliomas, with RF showing the best performance (AUC: 0.827) (25). Yamashita et al. used MRI images of 112 patients to predict the *TERT* promoter mutation status. The SVM model demonstrated good predictive performance (AUC: 0.776). The reported sensitivity, specificity, and accuracy were 0.85, 0.548, and 0.741, respectively (26). Furthermore, Joshi et al. employed a multi-classifier approach (including bagging, extra trees, random forest, gradient boosting, extra gradient boosting, and Adaboost) to stratify 135 patients with gliomas. Among all classifiers, the extra trees classifier achieved the highest average accuracy of 0.933. Additionally, within the ensemble-based classifiers, the bagging classifier, extra trees classifier, and random forest classifier also demonstrated favorable results (27). These studies suggest that selecting appropriate algorithms and combining multiple algorithmic models can help improve the diagnostic potential of different radiomics models, while also allowing for an intuitive comparison of their diagnostic performance. It is worth noting that the aforementioned studies focused solely on the analysis of low-grade gliomas or glioblastomas. Despite the inclusion of more feature selection and classification algorithms, there is a paucity of clinical research on the *TERT* promoter subtypes of grade 1–4 gliomas.

In a study by He et al. (28) involving 81 glioma cases, various models were constructed by combining clinical features with multiple sequences, including DWI and ADC. In the *TERT* genotype, the multi-sequence model incorporating all radiological features was found to better predict *TERT* status preoperatively. Additionally, Wang et al. (29) conducted a multi-modal MRI radiological study combining T1C, FLAIR, and ADC images. The automatically trained diagnostic model based on these diverse features exhibited a good ability to predict *TERT* promoter mutation type in gliomas. This indicates that utilizing models constructed from T1C, DWI, and ADC sequences to predict the *TERT* mutation status in glioma patients is a promising approach.

In the present study, the model built on RFE stood out in terms of diagnostic performance compared to other combinations. This suggests that the RFE feature selection method performs well in identifying the most relevant features and constructing predictive models, providing a more reliable tool for predicting *TERT* mutations in glioma patients. Additionally, models based on the combination of LR and LRLasso also exhibit good diagnostic performance. We found that contrast-enhanced T1-weighted imaging (T1CE) was the most important parameter in predicting the model and among the radiomic features in this study, the entropy of T1CE contributed the most to distinguishing between *TERT* mutated and *TERT* wild-type gliomas. The presence of contrast enhancement on T1-weighted scans is often considered a sign of malignancy in tumors because it indicates the presence of blood vessels within the tumor and can also reflect the size and location of the tumor. This can help inform preoperative treatment strategy (30, 31). Furthermore, the entropy of T1CE was found to be crucial for differentiating the *TERT* mutation status in glioma patients. As entropy can effectively capture the irregular distribution of grayscale levels and texture complexity in images, it helps in the extraction and quantification of the texture features of images, thereby enhancing the richness of image information (32, 33). Additionally, due to its favorable feature characteristics, frequency response, directional sensitivity, hierarchical structure, and dimensional properties that align with human vision, wavelet transformation has become an important tool for MRI image fusion and processing, with widespread applications (34). Unlike entropy, gray-level size zone matrix considers the connectivity between adjacent pixels and can calculate the number of connected regions of different gray levels and sizes. This allows for the description of intratumoral texture and structural features and the evaluation of tumor tissue heterogeneity (33). Therefore, entropy and gray-level size zone matrix are often used to evaluate tumor lesions. In other words, larger values of entropy and GLSZM indicate greater tumor heterogeneity, which may be attributed to increased complexity in the tumor microenvironment, including factors such as tumor vascular permeability and distribution of tumor cells (35). Therefore, entropy and GLSZM are useful markers and features for understanding tumor heterogeneity and complexity. Whether they can play a more profound role in the preoperative diagnosis and treatment of glioma patients remains to be discovered in larger research cohorts.

Our results show some interesting differences when compared to the existing literature. The 70% accuracy of the combined model we used (RFE + LRLasso) is 17.25% higher than the 52.75% accuracy of its SVC compared to the use of the conventional SVM model in the study of Navodini et al. (36). This result may provide richer feature information with our use of image data from multiple MRI sequences. In addition, we use multi-model combination, which makes our model more interpretable. In exploring multimodal data fusion, we found that Sasmitha et al. developed a machine learning model for analyzing brain tumors that combined MRI and WSI (whole slide imaging) data in a study to improve diagnostic efficiency (37). However, it also has some limitations, such as not explicitly mentioning the sample size, which may affect the reliability and generalization ability of the results. And relying on high-quality MRI and WSI data, when the image quality is not good enough, it does not ensure that their machine learning models can still have good analysis and diagnosis efficiency. In contrast, although our study only uses MRI data, the robustness and interpretability of the model are improved while ensuring the performance by combining multiple sequences and multiple models.

Some limitations of our study should be acknowledged. Firstly, our MRI image data were sourced from four different MRI scanners, which may have introduced an element of bias. Secondly, the analysis did not differentiate between glioma grades, such as high-grade and low-grade gliomas. However, covering patients with grades 1–4 gliomas is relatively more clinically relevant, as clinical settings may encounter patients with different grades of glioma. Additionally, some studies have utilized advanced imaging techniques, but due to their potentially higher cost and longer acquisition times compared to conventional imaging techniques, these imaging modalities may not be routinely used in practice, limiting their feasibility. Our study utilized routinely used conventional MRI sequences. Further studies are required to assess the predictive ability of novel functional MRI sequences and quantitative sequences for predicting glioma *TERT* promoter mutation status. Furthermore, since our study was retrospective, molecular profiling results (e.g., *IDH* mutations and *BRAF* mutations) were not completely consistent with histological grades. Therefore, we hope that in future research, radiomics technology will be applied to these molecular spectral analysis, so as to make the studied images more profound and meaningful.

## 5 Future directions and impact

Although this study has made remarkable progress in predicting the mutation status of *TERT* promoter in glioma, we think this is only the beginning. The application scope and influence of this method can be further expanded in the future. It is particularly noteworthy that our approach may play a greater role in dealing with tumors that are difficult to surgically remove. For example, diffuse midline gliomas (such as tumors located in the brainstem, thalamus, and spinal cord) often only have limited biopsy samples available, making comprehensive molecular testing difficult. In these cases, the ability to predict molecular features from MRI images will become particularly important, which may have a significant impact on treatment decisions. To achieve this goal, future research should focus on the following aspects: First, large-scale, multi-center validation studies are needed to ensure good applicability of our method in different populations and different types of brain tumors. Secondly, we should explore the combination of other imaging techniques (such as PET or advanced MRI sequences) or blood biomarkers to further improve the accuracy and reliability of prediction models. In addition, from the perspective of clinical application, our approach has the potential to promote the development of personalized treatment of brain tumors. Especially when routine molecular testing is not possible, this non-invasive predictive method may become a key tool in formulating treatment strategies. However, the incorporation of this predictive approach into clinical practice also requires ethical and regulatory challenges to be considered. Finally, we believe that future progress will require close cooperation between experts in the fields of radiology, neurosurgery, pathology, bioinformatics and artificial intelligence to promote the development and application of this technology, and ultimately improve the diagnosis and treatment effect of patients with brain tumors.

It is worth mentioning that we need to consider the application prospects of our model in future clinical settings. First, future work will focus on enhancing the accuracy and robustness of the proposed model. This can be achieved by integrating larger and more diverse datasets, which will help capture a wider range of tumor features and variants. In addition, integrating multi-omics data (such as genomics, transcriptomics, and proteomics) with radiogenomics signatures can provide a more comprehensive understanding of gliomas and their molecular subtypes. At the same time, an attempt should be made to develop advanced XAI technology to make the prediction of the model more transparent and easier for clinicians to understand. This includes generating visual explanations and providing insights into which features have the most impact on model decisions. User-friendly software is also available to provide clinicians with friendly conditions to easily enter patient data and build models. Finally, management should continually examine the application system of the model in clinical practice. It should also provide professional training and courses, create user manuals and guides, etc. While there are challenges in deploying AI-based models in clinical settings, the proposed model for predicting glioma *TERT* mutation status has great potential to enhance clinical decision-making. By addressing the outlined future work and deployment considerations, the model can be effectively translated into a valuable tool to improve patient outcomes for glioma treatment.

## 6 Conclusion

Using a combination of different feature selectors and classification algorithms may help predict glioma *TERT* promoter mutation status. In this study, models constructed using the LR and LRLasso algorithms showed the best predictive performance. Among the many features in radiomics, T1-weighted enhancement entropy and GLSZM demonstrated significant potential for non-invasive prediction of preoperative *TERT* promoter mutation status in glioma patients. These specific radiomic features can help tailor targeted and personalized treatment strategies for these patients.

## Data availability statement

The raw data supporting the conclusions of this article will be made available by the authors, without undue reservation.

## Ethics statement

The studies involving humans were approved by the Ethics Committee of Guangxi Medical University. The studies were conducted in accordance with the local legislation and institutional requirements. The ethics committee/institutional review board waived the requirement of written informed consent for participation from the participants or the participants’ legal guardians/next of kin because this is a retrospective study, so no informed consent is required.

## Author contributions

CT: Funding acquisition, Writing – original draft, Writing – review & editing. LC: Data curation, Formal analysis, Resources, Software, Writing – review & editing. YX: Investigation, Methodology, Project administration, Visualization, Writing – review & editing. LH: Funding acquisition, Resources, Supervision, Validation, Writing – review & editing. ZZ: Conceptualization, Project administration, Supervision, Writing – review & editing.

## Footnotes

^1^ https://www.slicer.org/
